# Futility in cancer surgery: towards consensus and patient-centred decisions

**DOI:** 10.1093/bjs/znag091

**Published:** 2026-09-21

**Authors:** Alison Wallace, Campbell S Roxburgh, Colin W Steele, John T Jenkins

**Affiliations:** St Marks Hospital, Complex Cancer Clinic, London, UK; School of Cancer Sciences, University of Glasgow, Glasgow, UK; School of Cancer Sciences, University of Glasgow, Glasgow, UK; School of Cancer Sciences, University of Glasgow, Glasgow, UK; St Marks Hospital, Complex Cancer Clinic, London, UK; School of Cancer Sciences, University of Glasgow, Glasgow, UK; Department of Surgery and Cancer, Imperial College London, London, UK

Futility in cancer surgery is commonly invoked as grounds to reject treatment yet remains poorly defined. It is not simply about whether an operation can be performed, but whether it is likely to deliver meaningful benefit to the patient in terms that matter to them. In the context of escalating treatment complexity, ageing populations, and growing pressure to justify high-risk surgery, the absence of agreed standards for futility can place both patients and clinicians in a difficult position^1^.

The problem is not that surgeons should never operate when benefit is uncertain. Rather, the term ‘futile’ is often used as if self-evident, when in reality it may refer to several distinct outcomes: intraoperative irresectability, early recurrence, short post-procedure survival, failure to receive adjuvant therapy, major morbidity, or unacceptable loss of quality of life. These endpoints describe different forms of treatment failure and should not be treated as interchangeable^2^.

Part of the difficulty is that futility contains both quantitative, predictable elements, and qualitative, individualized ones^3^. Some outcomes, particularly oncological ones such as survival, recurrence, or intraoperative irresectability, are relatively objective and increasingly amenable to prediction. Others, especially quality of life, functional independence, symptom burden, and the value a patient places on time gained, are far more subjective and cannot be reduced so easily to a standard threshold.

This distinction matters because binary global judgements of futility are often too crude for real clinical practice. An operation may be unlikely to deliver durable oncological benefit and yet still offer symptom relief or a period of meaningful time for a particular patient. Equally, a technically successful procedure may have little real value if it results in prolonged suffering, loss of independence, or rapid deterioration without meaningful survival gain. A treatment that is not worthwhile for one patient may still be acceptable to another with different goals and priorities. The central question is not simply whether an operation might fail on one metric, but whether it is likely to achieve the goals that matter most in that individual case.

This uncertainty has practical consequences. A recent high-profile disciplinary case involving the alleged offering of non-beneficial oncology treatment has highlighted the risks of acting without a shared framework for futility^4^. Whatever the details of such cases, they underline a broader truth: when clinicians, patients, and institutions do not agree on what counts as futile, decision-making becomes vulnerable to inconsistency, hindsight judgement, and moral distress.

These tensions are most apparent in advanced or metastatic disease, where multiple potentially valid treatment options exist and institutional preferences for certain approaches may be strong. The language of ‘aggressive’ *versus* ‘conservative’ surgery is unhelpful: most surgeons understandably wish to feel that they are doing as much as possible in their patients’ best interest, yet current literature offers little insight into how such preferences shape decisions about ‘futile’ *versus* ‘non-futile’ surgery, or how they intersect with patient priorities. More inclusive, methodologically robust studies, framed around explicit research questions and incorporating patient preference are required to clarify the real-world consequences of different treatment strategies in challenging settings such as oligometastatic or recurrent disease.

The existing literature does not yet resolve these questions. A recent review of predictive models for futile outcomes after curative cancer surgery identified wide heterogeneity in how futility is defined and a general lack of nuance about which facet of futility a given model is predicting. A model predicting postoperative morbidity rate is not predicting the same aspect of futility as one predicting death within 6 months, yet both may be discussed under the same label. Another recent systematic review of definitions of futility in advanced gastrointestinal cancers confirmed that most studies adopt a narrow oncological view, lack evidence of expert consensus and, notably, do not include patient perspectives^5^.

This lack of clarity is more than a minor methodological issue. If futility is defined arbitrarily, then a model may perform well statistically while still failing to answer the real clinical question: whether an operation should be offered at all. Most existing models reflect a relatively narrow oncological or quantitative view of futility, usually equating it with failure to cure, early recurrence, or short survival. Almost none address the more subjective dimensions of benefit that matter deeply to patients, particularly postoperative quality of life and functional outcomes. Beyond risk stratification, a better understanding of which patients derive no benefit from surgery could enable further study into the biological and phenotypic characteristics of such patients which in turn could help refine prediction tools and ultimately guide decision-making.

What is needed is a more disciplined and domain-specific approach. Rather than treating ‘futility’ as a monolith, the field should begin to define specific, clinically coherent domains for futility research—for example, short-term mortality and morbidity rates, early oncological failure, or unacceptable loss of function—recognizing that each captures a different way in which surgery may fail to deliver worthwhile benefit. Within each domain, definitions should be tailored by tumour type and clinical context, with explicit recognition that different facets of futility exist and should not be collapsed into a single binary judgement. Consensus-building work involving clinicians, patients, and, where appropriate, carers is needed so that definitions reflect real priorities rather than professional convenience. Predictive tools can then be developed and validated against outcomes that are transparent and directly meaningful within their domain.

Against this backdrop, the term ‘futility’ should be used with caution. It may be helpful as a prompt to pause, reflect, and reassess goals of care, but on its own it is an imprecise basis for choosing between complex treatment options. What is now required are broader, methodologically robust studies that are explicitly driven by carefully framed research questions, with embedded exploration of patient preferences. Such work is particularly pressing in settings of complex (that is oligometastatic and/or recurrent) disease, where practice is heterogeneous and choices are finely balanced. Building this evidence base and the language that accompanies it offers the best prospect of moving beyond ad-hoc judgements towards genuinely informed, patient-centred decisions about when cancer surgery is, and is not, likely to be worthwhile.
